# Chronic Intermittent Hypoxia Is Independently Associated with Reduced Postoperative Opioid Consumption in Bariatric Patients Suffering from Sleep-Disordered Breathing

**DOI:** 10.1371/journal.pone.0127809

**Published:** 2015-05-26

**Authors:** Alparslan Turan, Jing You, Cameron Egan, Alex Fu, Ashish Khanna, Yashar Eshraghi, Raktim Ghosh, Somnath Bose, Shahbaz Qavi, Lovkesh Arora, Daniel I. Sessler, Anthony G. Doufas

**Affiliations:** 1 Department of Outcomes Research, Cleveland Clinic, Cleveland, Ohio, United States of America; 2 Department of Quantitative Health Sciences, Cleveland Clinic, Cleveland, Ohio, United States of America; 3 Anesthesiology Institute, Cleveland Clinic, Cleveland, Ohio, United States of America; 4 Department of Anesthesiology, Perioperative and Pain Medicine, Stanford University School of Medicine, Stanford, California, United States of America; 5 Outcomes Research Consortium, Cleveland, Ohio, United States of America; Weill Cornell Medical College in Qatar, QATAR

## Abstract

**Background:**

Evidence suggests that recurrent nocturnal hypoxemia may affect pain response and/or the sensitivity to opioid analgesia. We tested the hypothesis that nocturnal hypoxemia, quantified by sleep time spent at an arterial saturation (SaO_2_) < 90% and minimum nocturnal SaO_2_ on polysomnography, are associated with decreased pain and reduced opioid consumption during the initial 72 postoperative hours in patients having laparoscopic bariatric surgery.

**Methods:**

With Institutional Review Board approval, we examined the records of all patients who underwent laparoscopic bariatric surgery between 2004 and 2010 and had an available nocturnal polysomnography study. We assessed the relationships between the time-weighted average of pain score and total opioid consumption during the initial 72 postoperative hours, and: (a) the percentage of total sleep time spent at SaO_2_ < 90%, (b) the minimum nocturnal SaO_2_, and (c) the number of apnea/hypopnea episodes per hour of sleep. We used multivariable regression models to adjust for both clinical and sleep-related confounders.

**Results:**

Two hundred eighteen patients were included in the analysis. Percentage of total sleep time spent at SaO_2_ < 90% was inversely associated with total postoperative opioid consumption; a 5-%- absolute increase in the former would relatively decrease median opioid consumption by 16% (98.75% CI: 2% to 28%, P = 0.006). However, the percentage of total sleep time spent at SaO_2_ < 90% was not associated with pain. The minimum nocturnal SaO_2_ was associated neither with total postoperative opioid consumption nor with pain. In addition, neither pain nor total opioid consumption was significantly associated with the number of apnea/hypopnea episodes per hour of sleep.

**Conclusions:**

Preoperative nocturnal intermittent hypoxia may enhance sensitivity to opioids.

## Introduction

Sleep disruption and recurrent nocturnal hypoxemia are distinct pathophysiological components of obstructive sleep apnea (OSA), and both are thought to influence pain processing [1–3] and opioid analgesia [4,5]. Experimental sleep fragmentation and sleep deprivation enhance sensitivity to pain [1,2], promote inflammation [6], and augment spontaneous pain [7] in healthy humans. As one might thus expect, patients with insomnia [8] and patients with temporomandibular joint disorder who suffer from primary insomnia [9], demonstrate hyperalgesia. In striking contrast, patients with temporomandibular joint pain and OSA are hypoalgesic to experimental pain [9].

The contrast between insomnia and OSA with respect to pain sensitivity suggests that the two basic phenotypic components of OSA namely sleep disruption and recurrent nocturnal hypoxemia, have different or even opposite effects on pain processing. Consistent with this hypothesis, there is an association in OSA patients between nocturnal intermittent hypoxia and increased analgesic sensitivity to opioids [5]. Furthermore, children with recurrent nocturnal hypoxemia due to OSA are more sensitive to the analgesic effect of morphine; specifically, children with a nadir oxyhemoglobin saturation (SaO_2_) < 85% required half the dose of morphine after adenotonsillectomy than those with a nadir SaO_2_ ≥ 85% [4].

Better understanding of the relationship between OSA, pain, and opioid toxicity may facilitate perioperative opioid titration and improve analgesic safety in this vulnerable patient population. We thus examined postoperative pain and opioid consumption in patients with OSA who had laparoscopic weight-loss surgery. Specifically, we tested the hypothesis that nocturnal intermittent hypoxia in the context of OSA, as quantified by sleep time spent at a SaO_2_ < 90% and minimum nocturnal SaO_2_, as well as the number of apnea /hypopnea episodes during sleep, are associated with decreased pain and reduced opioid consumption during the initial 72 postoperative hours in patients having bariatric surgery.

## Materials and Methods

### Patient selection

With Cleveland Clinic Institutional Review Board approval, written informed consent was waived for this retrospective cohort analysis of 1641 adults who underwent laparoscopic bariatric surgery at the Cleveland Clinic main campus between 2004 and 2010. First, we manually searched all individual charts to identify those patients who had a sleep study performed within two preoperative years. Consequently, we inquired the results of the sleep studies (polysomnography reports) and focused on collecting information regarding the polysomnography parameters and other predetermined variables, required for the proposed analysis. Patients were excluded from the analysis based on the unavailability of data in the nocturnal oxygenation, opioid consumption, and /or confounding variables.

### Outcomes and Exposures

Opioid consumption and pain perception (measured by numerical pain rating scale) in the first 72 hours postoperatively, were the main outcomes of our analysis. Explanatory variables were the exposure to chronic intermittent hypoxia as measured by the polysomnography oxygenation parameters (i.e., sleep time spent at a SaO_2_ < 90% expressed as a percentage of total sleep time, and minimum nocturnal SaO_2_), as well as the number of apnea /hypopnea episodes per hour of sleep (i.e., the apnea /hypopnea index).

### Anaesthetic Management

All patients received the same anaesthetic management, which did not include epidural anaesthesia. More specifically, induction to general anaesthesia was performed with propofol, fentanyl, and rocuronium (and /or succinylcholine), while sevoflurane or isoflurane in oxygen /air mixture, were used for anaesthetic maintenance. Intraoperative analgesia by fentanyl and hydromorphone was titrated to patients’ vital signs. Abdominal port sites for the laparoscopic instruments were infiltrated with local anesthetics.

#### Postoperative pain management

After surgery, patients arrived at the post-anaesthesia care unit (PACU), were evaluated by nurses and, if required, they received analgesic medications in accordance to the attending anesthesiologist’s orders. At the Clinic, fentanyl, morphine, and /or hydromorphone are the drugs used most often in that setting. Patients were given incremental doses of the prescribed opioids to keep pain scores (numerical raring scale: 0–10; with 10 indicating worst pain) below 4; vital signs monitoring included electrocardiography, heart rate, non-invasive blood pressure, and pulse oximetry. Oxygen administration ensures a SaO_2_ > 90%.

After transfer to a ward, patient-controled analgesia (PCA) is the preferred method of administering opioids in the immediate and short-term postoperative period. Hydromorphone is the primary agent used in that setting (with a baseline infusion set at 0, a bolus dose of 0.2–0.4 mg, and a lockout interval set at 6–10 min), with morphine (0 / 1–2 mg / 6–10 min) as an alternative, and fentanyl (0 / 20 mcg / 6–10 min) in case of a history of allergic or other type of reaction to the previous two. When patients start tolerating oral medications, oxycodone 5–10 mg /acetaminophen 325 mg, oxycodone 5–10 mg, or hydromorphone 2–4 mg, are administered every 4 hours. A short period exists when both the intravenous and oral route of opioid administration are used, to ensure that patients’ analgesia is maintained during the transition. Pain is evaluated every 4 hours using numerical rating scale, throughout the postoperative period.

According to Clinic’s policy, patients, who use continuous positive airway pressure (CPAP) for OSA, are encouraged to use this treatment in the postoperative period.

### Measurements

#### Polysomnography

All patients had an attended nocturnal polysomnography study evaluation in a clinical sleep laboratory facility. Sleep studies were performed and evaluated, according to consensus and practice guidelines published by the American Academy of Sleep Medicine [10–13]. Nocturnal polysomnography evaluation reports were recovered from electronic records or patient charts.

We collected data on two polysomnography parameters that well reflect nocturnal oxygenation during sleep and have been shown in the past to be independent risk markers for cardiometabolic morbidity [14–17] in patients suffering from sleep-disordered breathing: (a) percentage of total sleep time spent at a SaO_2_ < 90%; and, (b) minimum nocturnal SaO_2_ during sleep. The number of apnea /hypopnea episodes per hour of sleep (i.e., apnea /hypopnea index) was also noted.

We have also collected data on the following five polysomnography parameters reflecting patterns of fragmented and /or inadequate sleep, which has been implicated in enhancing sensitivity to pain in experimental and clinical settings [18]: (a) sleep efficiency, calculated as the percentage of the time in bed that was actually spent asleep; (b) the number of awakenings, defined as electroencephalographically detected arousals lasting more than 15 sec; (c) time spent awake after sleep onset; (d) number of sleep stage shifts; and, (e) cumulative duration of sleep stages 3 and 4, as % of total sleep time. Data on these polysomnography parameters were used in the analysis as potential confounders of the relationship between nocturnal intermittent hypoxia and opioid consumption or pain perception.

#### Perioperative data collection

Cleveland Clinic Perioperative Health Documentation System and paper charts were used to recover patient information including age, gender, race, body mass index, smoking status, and comorbidities including diabetes mellitus, chronic pain disorder, chronic use of steroids and /or opioids. Pain scores (0-10-point numerical rating scale; with 10 indicating worst pain) were normally obtained at 15-minute-intervals in the PACU and every 4 hours on the surgical ward. We also determined the doses of analgesic medications administered to the patients during the initial 72 postoperative hours. Data were collected from electronic medical records, and the total amount of opioids used was converted to morphine equivalents for the analysis. For this conversion we employed a conversion table, which was constructed after surveying the relevant literature [19,20] for each individual opioid and has been previously used in retrospective [21] and prospective [22] clinical trials.

### Statistical Analysis

Our analysis focused on the association between our primary outcomes of pain score and opioid consumption in the 72 hours after surgery and polysomnography parameters reflecting nocturnal oxygenation and apnea /hypopnea status during sleep.

We assessed the relationships between the time-weighted average pain score and total opioid consumption (after logarithm transformation for meeting the normality modeling assumption) during the first 72 hours after surgery, and: (a) the percentage of total sleep time spent at SaO_2_ < 90% and (b) the minimum nocturnal SaO_2_ (a total of 4 analyses), each using a multivariable regression model. The difference in mean time-weighted average pain score and the ratios of medians in opioid consumption (obtained by back-transforming the difference in means on the log scale) for 5-%-absolute increase in the percentage of total sleep time spent at SaO2 **<** 90% and 1-%-absolute decrease in the minimum nocturnal SaO_2_, with confidence intervals (CI), were reported. The time-weighted average of pain score is equal to the sum of the portion of each time interval in-between two adjacent pain score measurements multiplied by the average of the corresponding two pain scores and divided by the time interval between the first and the last pain score measurements.

We pre-specified the following 16 potential confounders including age, gender, race, body mass index, smoking status, diabetes mellitus, chronic pain syndrome, chronic use of systemic steroids and opioids, continuous positive airway pressure (CPAP) therapy, sleep efficiency, number of awakenings, time spent awake after sleep onset, number of sleep stage shifts, percent of total sleep time spent in stages 3 and 4, and type of bariatric surgery (i.e., laparoscopic gastroenterostomy vs. others). All the above potential confounders were considered for including in the model through the use of a backward selection procedure (alpha-to-stay was set conservative at 0.20). In addition, we conducted a sensitivity analysis for the total opioid consumption and time-weighted average of pain score for the time period from 6 to 72 hours after surgery, using the same analysis method. A Bonferroni correction was used to adjust for multiple testing; the significance criterion for each analysis was P < 0.0125 (i.e., 0.05 /4).

Secondly, we aimed to estimate the associations between the time-weighted average of pain score and total opioid consumption (after logarithm transformation), and: (a) the apnea /hypopnea index, and (b) a formal diagnosis of OSA using the same method as in the above analyses. The significance criterion for each secondary analysis was P < 0.0125 (i.e., 0.05 /4).

With 218 available patients, we had more than 90% power to detect the following treatment effects at the overall significance level of 0.05 (criterion of 0.0125 for each primary analysis). Every 5-%- absolute increase in the percentage of total sleep time spent at SaO_2_ < 90% would be associated with: (a) a change (increase or decrease) of 0.125 in the time-weighted average of pain score (i.e., a slope of ± 0.025), and (b) a relative increase of 30% (i.e., a slope of 0.075 on log scale) or a relative decrease of 23% (i.e., a slope of -0.075 on log scale) in median opioid consumption. Every 1-%- absolute decrease in the minimum nocturnal SaO_2_ would be associated with: (a) a change (increase or decrease) of 0.05 in the time-weighted average of pain score (i.e., a slope of ± 0.05), and (b) a relative increase of 11% (i.e., a slope of 0.15 on log scale) or a relative decrease of 10% (i.e., a slope of -0.15 on log scale) in median opioid consumption. We assumed a standard deviation (SD) of 20% for the percentage of total sleep time spent at SaO_2_ < 90%, a SD of 10 for the minimum nocturnal SaO_2_, a SD of 2 for the time-weighted average of pain score, and a SD of 6 for the total opioid consumption (on the log scale).

R function power.SLR (package powerMediation) in version 2.15.3 (The R Foundation for Statistical Computing, Vienna, Austria) was used for power analysis. The Statistical Analysis System (SAS Institute, Cary, NC, USA) software version 9.3 was used for all the other analyses.

## Results

**Fig 1:**
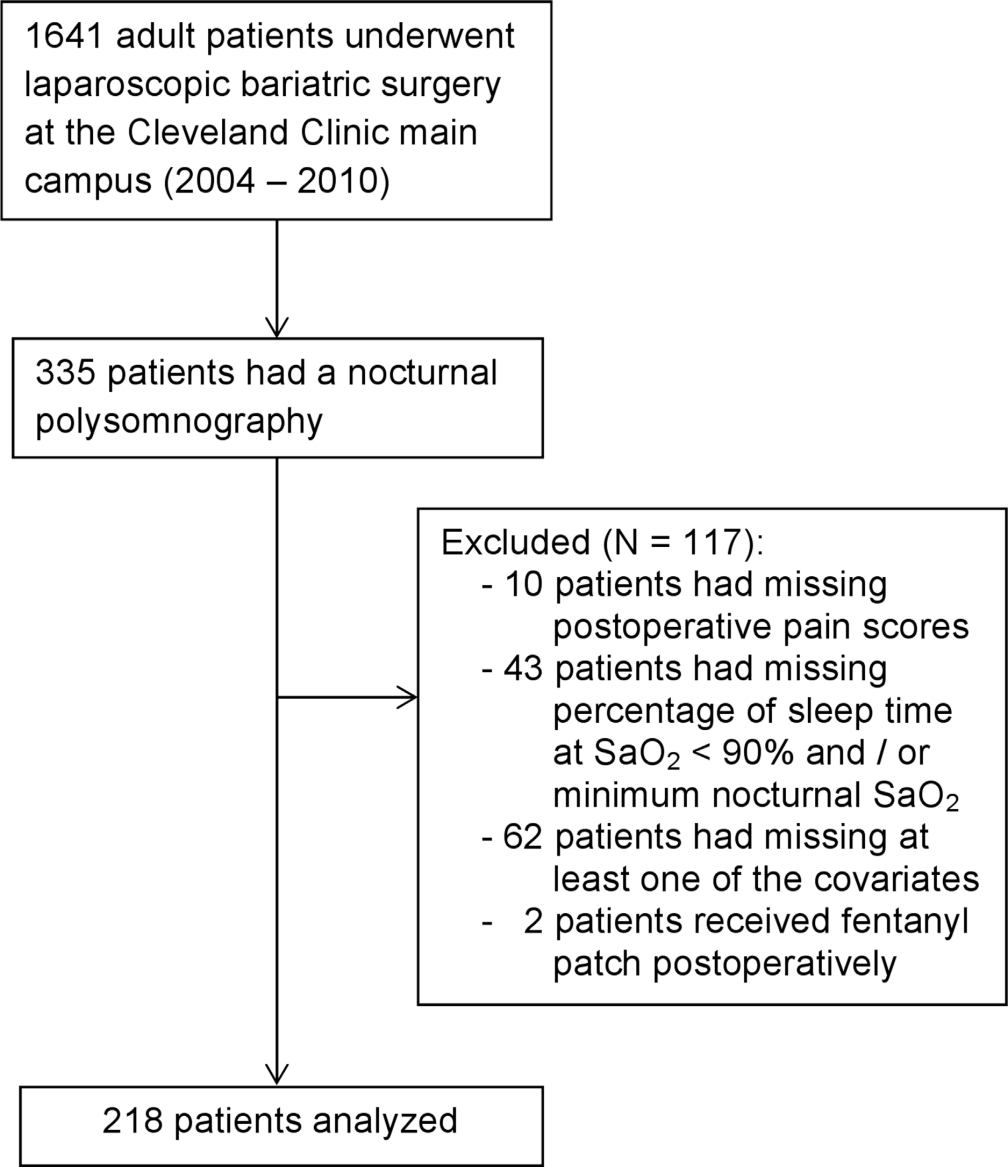
Flow diagram for the selection of eligible cases.

A total of 218 patients were included in our primary analysis. A screening log for our cohort is presented in Fig 1. From these 218 eligible patients, 132 (61%) presented with moderate-to-severe OSA as documented by an apnea /hypopnea index (AHI) ≥ 15 events per hour of sleep. All procedures were carried out by laparoscopy and included Roux-en-Y gastric bypass, laparoscopic adjustable gastric band, and sleeve gastrectomy (Table 1).

**Table 1:** Morphometrics, clinical characteristics, and polysomnography variables (N = 218*).

Variable	Statistics ^†^
Age, years	47 ± 11
Gender (female), no. %	157 (72)
Race, no. %	
Caucasian	169 (77.5)
African American	41 (18.8)
Others	8 (3.7)
Body mass index, kg/m^2^	46 [42 to 52]
Smoking status (smokers), no. %	105 (48)
Diabetes, no. %	74 (34)
Chronic systemic steroid use, no. %	10 (5)
Chronic opioid use, no. %	55 (25)
Chronic pain syndrome, no. %	139 (64)
Obstructive sleep apnea	191 (88%)
Continuous positive airway pressure therapy, no. (%)	138 (63)
Type of gastric surgery ^#^, no. %	
Gastroenterostomy	178 (82)
Gastric restrictive procedure	36 (17)
Gastroplasty	3 (1)
Removal of gastric restrictive device	1 (<1)
Polysomnography parameters	
Sleep efficiency, no. %	78 ± 14
Number of awakenings	23 [17 to 33]
Wake time after sleep onset, minutes	57 [31 to 90]
Number of sleep stage shifts	110 [80 to 145]
Sleep time spent in stages 3 and 4, % of TST	9.8 [1.4 to 14.7]
Apnea Hypopnea Index (AHI) ^‡^, no. (%)	
AHI < 5	32 (15)
5 ≤ AHI < 15	51 (24)
15 ≤ AHI < 30	47 (22)
AHI ≥ 30	85 (40)
Time spent at SaO_2_ < 90%, % of TST	7.8 [1.1 to 26.0]
Minimum nocturnal SaO_2_, %	82 [74 to 86]

* Out of 335 patients who had nocturnal polysomnography, 115 patients with missing total sleep time SaO_2_ < 90%, minimum nocturnal SaO_2_, and /or other covariates, and 2 patients received fentanyl patch, were excluded from the primary analysis.

^†^ Statistics are reported as mean ± SD, median [1^st^, 3^rd^ quartile], or No. (%).

^#^ Type of gastric surgery was categorized based on The International Classification of Diseases, Ninth Revision, Clinical Modification (ICD-9) code

Gastroenterostomy (ICD-9:44.38)
Bypass: gastroduodenostomy Gastroenterostomy Gastrogastrostomy Laparoscopic gastrojejunostomy without gastrectomy NEC
Gastric restrictive procedure (ICD-9: 44.95) Adjustable gastric band and port insertion Gastroplasty (ICD-9: 44.68) Banding Silastic vertical banding Vertical banded gastroplasty Code also any synchronous laparoscopic gastroenterostomy (44.38) Removal of gastric restrictive device(s) (ICD-9: 44.97) Removal of either or both: adjustable gastric band and subcutaneous port device

^‡^ Three patients had missing AHI value

SaO_2_: arterial oxygen saturation by pulse oximetry; TST: total sleep time.

Intraoperative analgesia was accomplished by fentanyl, hydromorphone, morphine, alfentanil, remifentanil, and hydromorphone, while 15 patients were also received ketorolac. Dexamethasone was administered intraoperatively in 89 patients. Postoperatively, intravenous (fentanyl, hydromorphone, and morphine) and oral (hydrocodone, hydromorphone, meperidine, oxycodone, oxycodone /acetaminophen, and tramadol) opioids were used to treat pain. The majority of the patients received intravenous patient-controlled analgesia with fentanyl (162, 74%), while patient-controlled analgesia with hydromorphone (32, 15%) and morphine (22, 10%) were also used. Only a small number of patients received non-opioid analgesics, including acetaminophen (N = 9), ketorolac (N = 3), and aspirin (N = 1).

**Fig 2:**
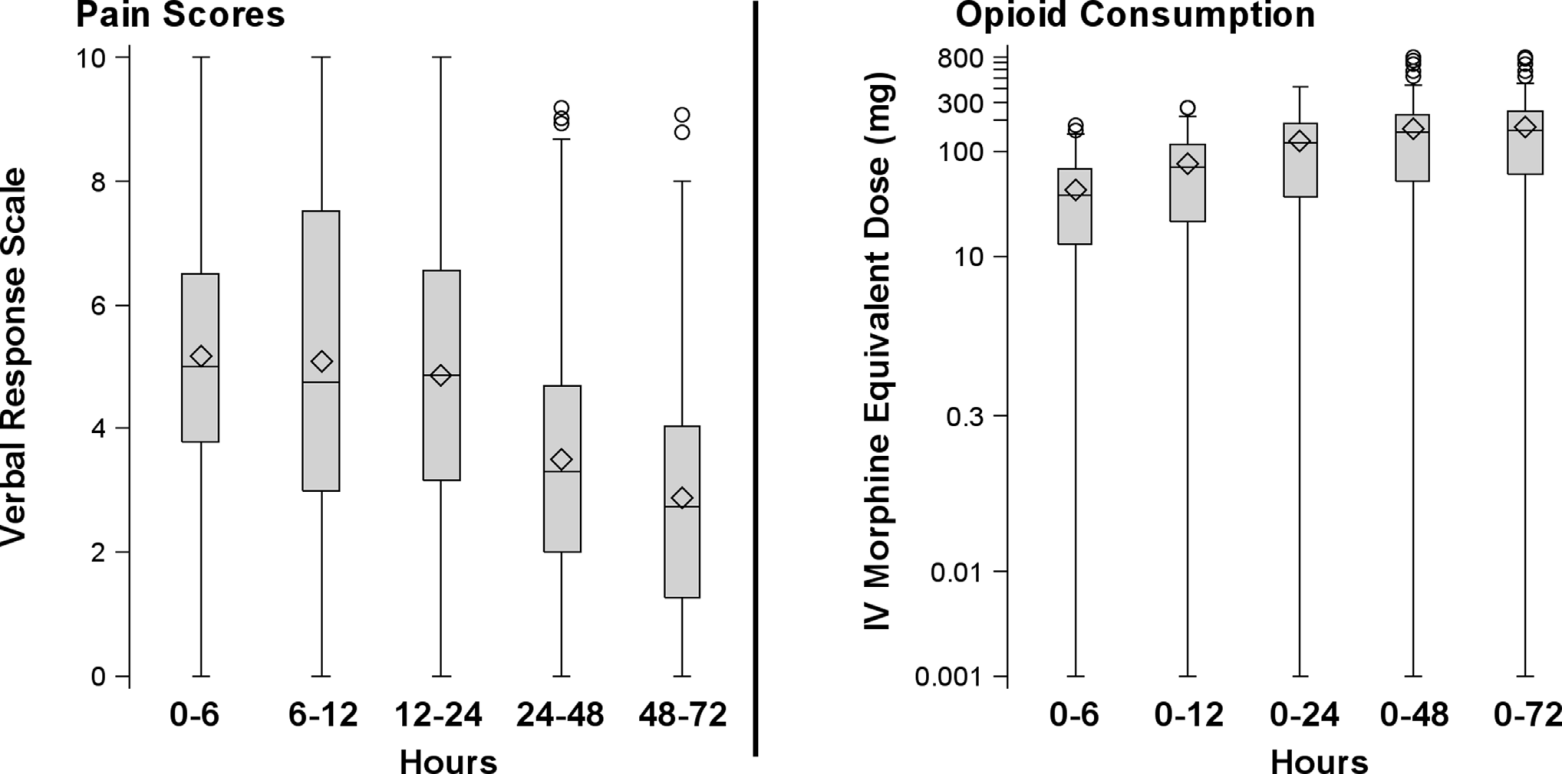
Boxplots of time-weighted average pain scores (11-point verbal numerical rating scale; 0–10) and of total opioid consumption in mg of intravenous morphine equivalent dose obtained at 4 discrete postoperative intervals (0–12, 1–24, 0–48, and 0–72 hours after surgery) for the 218 patients included in the analysis. The first quartile, median, and third quartile comprise the boxes; whiskers extend to the most extreme observations within 1.5 times the interquartile range of the first and third quartiles, respectively; points outsides these whiskers are displayed individually. IV = intravenous.

**Fig 3:**
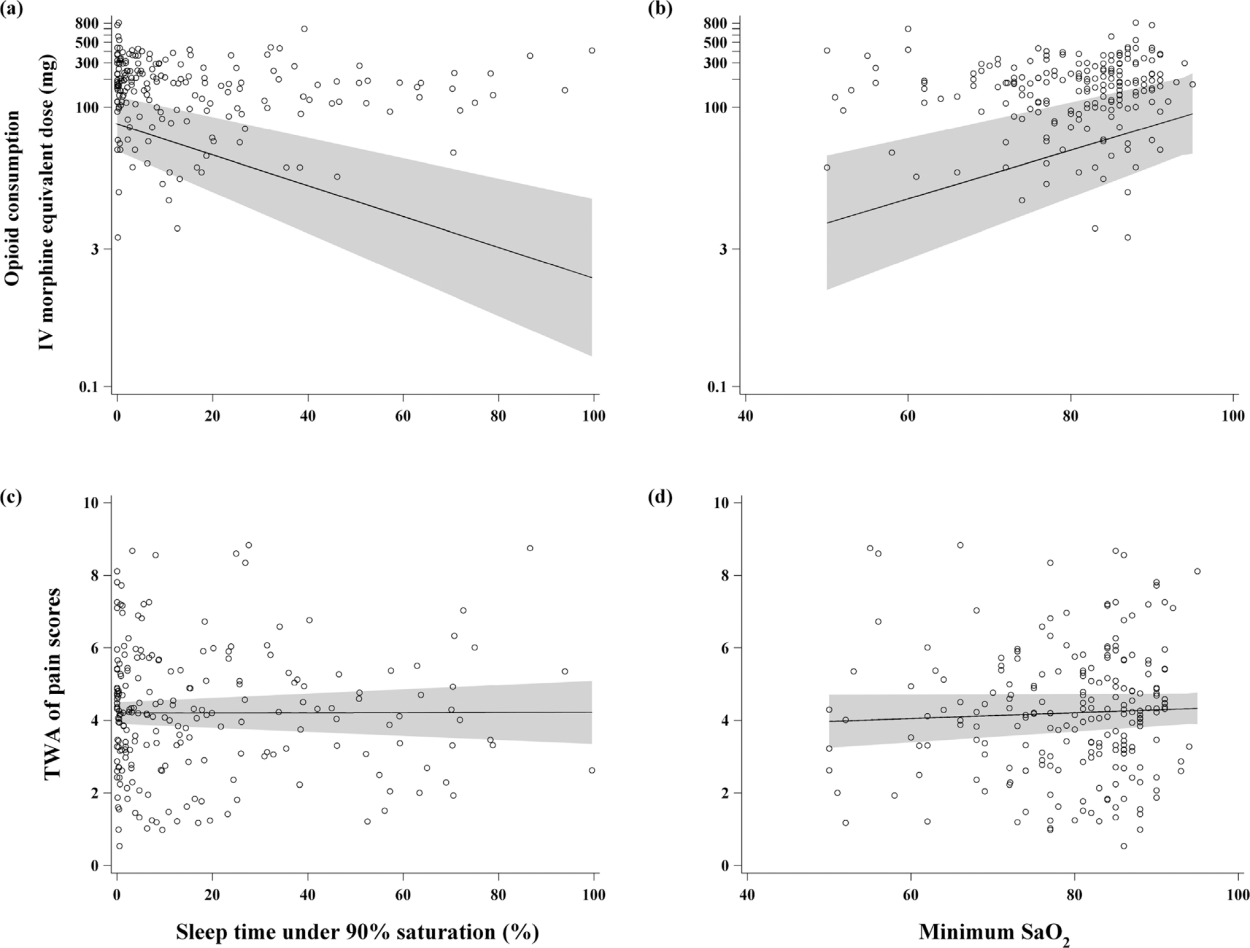
Upper graphs show the scatter plots of total opioid consumption (on log-scale) during the initial 72 postoperative hours versus the percentage of total sleep time spent at SaO_2_ < 90% (a), and the minimum nocturnal SaO_2_ (b). Lower graphs show the scatter plots of time-weighted average (TWA) of pain score versus the percentage of total sleep time spent at SaO_**2**_ < 90% (c), and the minimum nocturnal SaO_**2**_ (d). All the associations are unadjusted. Solid lines are the fit regression lines and shaded regions represent the corresponding 95% confidence bands. IV = intravenous; SaO_**2 =**_ arterial oxygen saturation by pulse; TWA = time-weighted average.

Twenty five (11%) and 94 (43%) patients were discharged on postoperative day 1 and 2, and 99 (45%) patients were discharged on postoperative day 3 or later, respectively. Up to the first 72 hours postoperatively, the observed mean (SD) of the patient-specific time-weighted average of pain score (numerical rating scale) was 4.2 (1.7), and the median opioid consumption (in morphine equivalents) was 158 mg [Q1, Q3: 61 to 246], (Fig 2). The observed medians for the percentage of sleep time spent at SaO_2_ < 90% and minimum nocturnal SaO_2_ in the preoperative polysomnography study, were 7.8% (1.1 to 26.0) and 82% (74 to 86), respectively (S1 Fig). The unadjusted associations are also presented in Fig 3.

Total opioid consumption during the first 72 postoperative hours was inversely associated with sleep time spent at SaO_2_ < 90% (P = 0.006). The adjusted estimated ratio of medians of total opioid consumption was 0.84 (98.75% CI: 0.72 to 0.98) for a 5-%-absolute increase in the percentage of total sleep time spent at SaO_2_ < 90% (Table 2). That means, the estimated median postoperative opioid consumption would relatively decrease by 16% (i.e., 100%- 100% × 0.84) for each 5-%-absolute increase in the percentage of total sleep time spent at SaO_2_ < 90%, by 29% (i.e., 100%- 100% × 0.84 × 0.84) for a 10-%-absolute increase, and so forth. However, the time-weighted average of pain score during the same postoperative period was not associated with the total sleep time spent at SaO_2_ < 90% (P = 0.18). The adjusted increase in the time-weighted average of pain score was 0.04 (98.75% CI: -0.03 to 0.10) for a 5-%-absolute increase in the percentage of total sleep time spent at SaO_2_ < 90%. The sensitivity analysis provided consistent results (Table 2). These associations were consistent across the different types of surgery (exposure—by—type interaction P—value were 0.11 and 0.91, respectively).

**Table 2:** Primary results for the nocturnal arterial saturation status. *(N = 218).

Outcome / Explanatory variable	Ratio of medians (98.75% CI)^#^	P-value^#^
Total opioid consumption ^¶^ / Percentage of sleep time spent at SaO_2_<90% (per 5-%-absolute increase)	Unadjusted: 0.83 (0.71, 0.96)	0.001
	Adjusted^†^: 0.84 (0.72, 0.98)	0.006
	Sensitivity^$^: 0.82 (0.68, 1.00)	0.01
Total opioid consumption ^¶^ / Minimum nocturnal SaO_2_ / (per 1-%-absolute decrease)	Unadjusted: 0.94 (0.88, 1.01)	0.03
	Adjusted^†^: 0.94 (0.88, 1.01)	0.03
	Sensitivity^$^: 0.93 (0.86, 1.02)	0.04
	**Mean difference (98.75% CI)**	
TWA of pain scores / Percentage of sleep time spent at SaO_2_<90% (per 5-%-absolute increase)	Unadjusted: 0.00 (-0.06, 0.07)	0.97
	Adjusted^‡^: 0.04 (-0.03, 0.10)	0.18
	Sensitivity^$^: 0.03 (-0.04, 0.10)	0.32
TWA of pain scores / Minimum nocturnal SaO_2_ (per 1-%-absolute decrease)	Unadjusted: -0.01 (-0.04, 0.02)	0.51
	Adjusted^‡^: 0.02 (-0.01, 0.05)	0.17
	Sensitivity^$^: 0.01 (-0.02, 0.05)	0.35

* Linear regression model was used.

^¶^ Total opioid consumption was analyzed after logarithm transformation for meeting the normality modeling assumption. The estimated ratio of medians in opioid consumption was obtained by back-transformation the difference in means on the log scale.

^#^ A Bonferroni correction was used to adjust for multiple testing; the significance criterion for each individual analysis was P < 0.0125 (i.e., 0.05 /4). Thus, 98.75% confidence intervals are reported.

The following variables were retained in the model via the backward selection procedure:

^†^ smoking, chronic pain syndrome, chronic use of systemic steroids and opioids, number of awakenings, and type of bariatric surgery;

^‡^ age, gender, chronic usage of steroids, chronic pain syndrome, sleep efficiency, and time spent awake after sleep onset.

^$^ Sensitivity analyses: we included pain scores and opioid consumption from 6 to 72 hours after surgery. The same set of covariates was considered for inclusion in each analysis.

SaO_2_: arterial oxy-hemoglobin saturation; TWA: time-weighted average.

Neither total opioid consumption (P = 0.03 > significance criterion of 0.0125, Bonferroni correction) nor time-weighted average of pain score (P = 0.17) was associated with the minimum nocturnal SaO_2_, during the first 72 hours after surgery. Consistent results were obtained from our sensitivity analysis (Table 2). Similarly, no interaction with type of surgery was observed (P-value = 0.78 and 0.42 for the above two analyses, respectively).

There were 119 (55%) patients who were discharged on postoperative day 1 or 2. Among these patients, 32 (27%) had no pain at discharge (pain score of 0), 45 (38%) had mild pain (pain score 1 to 3), 31 (26%) had moderate pain (pain score 4 to 6), and 11 (9%) had severe pain (pain score 7 to 9) at discharge, respectively. In order to examine the possibility of underestimating the total opioid consumption during the first 72 hours postoperatively (especially for the 11 patients who had severe pain at discharge), we conducted a sensitivity analysis where we used the total opioids consumption up to 48 hours as the outcome. We found that the estimated ratio of medians of total opioid consumption was 0.84 (0.72 to 0.98) for a 5-%-absolute increase in the percentage of total sleep time spent at SaO2 < 90% (P = 0.005) and 0.94 (0.88 to 1.01) for a 1-%-absolute decrease in the minimum nocturnal SaO_2_ (P = 0.03). These results are almost identical with the results of our primary analysis.

Secondly, we found that neither time-weighted average of pain score nor total opioid consumption was significantly associated with apnea /hypopnea index or a preoperative diagnosis of OSA (Table 3).

**Table 3:** Secondary results for the apnea /hypopnea index and OSA diagnosis *(N = 218).

Outcome	Explanatory variable	Ratio of medians (98.75% CI)^#^	P-value^#^
Total opioid consumption ^¶^	AHI (per 5-event increase)	0.93 (0.84 to 1.04) ^a^	0.12
	OSA (yes versus no)	0.23 (0.03 to 2.01) ^b^	0.09
		**Mean difference (98.75% CI)** ^**#**^	
TWA of pain scores	AHI (per 5-event increase)	0.01 (-0.04 to 0.06) ^c^	0.54
	OSA (yes minus no)	-0.45 (-1.40 to 0.49) ^d^	0.23

* Linear regression model was used.

^¶^ Total opioid consumption was analyzed after logarithm transformation for meeting the normality modeling assumption. The estimated ratio of medians in opioid consumption was obtained by back-transformation the difference in means on the log scale.

^#^ A Bonferroni correction was used to adjust for multiple testing; the significance criterion for each individual analysis was P < 0.0125 (i.e., 0.05 /4). Thus, 98.75% confidence intervals are reported.

The following variables were retained in the model via the backward selection procedure

^a^ age, smoking chronic pain syndrome, chronic usage of opioids, time spent awake after sleep onset, percent of total sleep time spent in stages 3 and 4, and type of bariatric surgery;

^b^ smoking, chronic pain syndrome, chronic usage of steroids and opioids, time spent awake after sleep onset, percent of total sleep time spent in stages 3 and 4, and type of bariatric surgery;

^c^ age, gender, chronic pain syndrome, chronic usage of steroids, sleep efficiency, and time spent awake after sleep onset; and

^d^ age, gender, smoking, chronic pain syndrome, chronic usage of steroids, sleep efficiency, and time spent awake after sleep onset.

AHI: apnea /hypopnea index; OSA: obstructive sleep apnea; TWA: time-weighted average.

## Discussion

We found that total opioid consumption in the first 72 hours after laparoscopic bariatric surgery was significantly associated with nocturnal intermittent hypoxia (measured by the total sleep time spent at SaO_2_ < 90%) in patients suffering from OSA. Patients with longer arterial desaturations in polysomnography consumed significantly less opioid for postoperative pain management.

Our regression model was adjusted for the presence of sleep fragmentation in polysomnography, the use of continuous positive airway pressure, and other factors with a potential influence on a patients’ response to pain and/or opioid analgesia. We selected arterial desaturation indices as explanatory variables based on *a priori* information linking intermittent hypoxia to increased opioid sensitivity and is consistent with current trends in OSA outcomes research, which is shifting focus from the crude measure of apnea /hypopnea index [23] to more physiologic indices of oxygen desaturation and electroencephalography-detected arousals from sleep that are potentially better markers for OSA-related morbidity. As expected, we did not find a significant association between either postoperative pain score or opioid consumption and apnea /hypopnea index or a formal diagnosis of OSA, in our patient population. This finding is in agreement with the results of a recent prospective investigation in general surgery patients, which failed to show an association between preoperative apnea /hypopnea index and postoperative opioid consumption in patients suffering from OSA [24].

Attended preoperative polysomnography showed that approximately 60% of our bariatric patients were suffering from moderate-to-severe OSA (apnea /hypopnea index ≥ 15 events per hour of sleep), which agrees with the current epidemiological profile of the disease in this age and BMI range [25]. A median nocturnal nadir SaO_2_ of 82% and a 7.8% of sleep time spent at a SaO_2_ < 90% indicate clinically important hypoxemia, which compares with magnitudes of nocturnal hypoxemia in studies establishing OSA as an independent risk factor for stroke (median % of total sleep time at SaO_2_ < 90%: 0.4 vs. 0.1, with and without ischaemic stroke), [14]; cardiovascular disease (hypopneas with a 4% or more decrease in SaO_2_ were predictive vs. those with less 4%), [15]; atrial fibrillation (each 1-%-absolute decrease in the mean nocturnal SaO_2_ tripled the risk for atrial fibrilation), [16]; insulin resistance (average nocturnal SaO_2_ < 94% and more than 2.17% of total sleep time at SaO_2_ < 90% predicted insulin resistance), [17]; and sleep-related pain (a decrease in the minimum nocturnal SaO_2_ from 92 to 75% approximately doubled the odds for pain), [3].

Our findings support previous evidence from both the clinical [4,26] and experimental [5,27,28] domain, in which recurrent nocturnal arterial desaturation has been shown to increase sensitivity to the analgesic effects of opioids. More specifically, in children having adenotonsillectomy, the morphine requirement for postoperative analgesia was inversely related to the amount of preoperative nocturnal arterial desaturation [4,26]. Although Brown et al., [4,26] used nocturnal continuous oximetry monitoring rather than polysomnography, their findings were supported by independent experiments showing that intermittent hypoxia up-regulates μ-opioid receptors in developing rats [27,28] and may thus be responsible for increased sensitivity to the analgesic and respiratory effects of opioids [29–31]. Consistent with these findings, we have also shown that nocturnal hypoxemia (expressed by minimum nocturnal SaO_2_ in polysomnography) in adults suffering from OSA, is associated with an increased analgesic sensitivity to short-acting opioids [5]. Furthermore, insulin growth factor binding protein-1 (IGFBP-1), a serum marker of hypoxia[32], is associated with hypoalgesia to experimental heat, as well as increased potency of opioid analgesia [5].

Seemingly in contrast to our findings, a prospective study cohort evaluating the effect of race on postoperative pain in children undergoing adenotonsillectomy, showed that African American children with OSA required more opioids for pain management and experienced longer post-anaesthetic recovery due to inadequate pain control than Caucasian children suffering from OSA. Interestingly, Caucasian children (with or without OSA) experienced a higher incidence of opioid-related side effects [33]. But because the diagnosis of OSA in these studies was based upon an abnormal apnea /hypopnea index which might not correlate with either nocturnal oxygenation or sleep quality status, the potential relationship between nocturnal intermittent hypoxia and postoperative pain and /or opioid consumption remains uncertain.

Collectively, clinical [4,26,33] and experimental [5,9,34] findings support the theory that OSA affect pain perception and analgesic sensitivity to opioids through at least one pathway related to chronic intermittent hypoxia. However, establishing nocturnal recurrent hypoxemia as an independent risk marker for increased pain and /or postoperative opioid consumption in OSA patients will require an intense effort in the context of well-controlled prospective cohorts since a plethora of potential confounders, including sleep quality, inflammation, and genetic predisposition may dilute the isolated effect of hypoxia.

The bariatric patients included in our analysis had a nocturnal polysomnography evaluation performed in the past and, presumably, all carried certain risk markers for OSA (e.g., increased body mass). This might have reduced a potential bias associated with withholding opioids for postoperative pain management in patients with certain OSA-related phenotypes. But it is also possible that OSA patients with more severe arterial desaturations in the nocturnal polysomnography were more sensitive to the sedative effect of opioids and, compounded with the residual effect of general anaesthetics, more likely to present with airway obstruction and /or desaturation events in the PACU. However, we believe that the potential for such a bias in our analysis is rather minimal because: a) opioid administration at the Clinic is titrated to analgesia, defined by a specific pain score, making dose adjustments in response to baseline characteristics less likely, b) symptoms of excessive daytime sleepiness in OSA patients, even in the presence of nocturnal hypoxemia, have been associated with the sleep fragmentation and chronic sleep deprivation phenotypes of the disease (for the presence of which our analysis is adjusted) rather, than the severity of arterial desaturations per se [35,36], c) in the first 6 hours after surgery, patients used about 25% of the total amount of opioids consumed in the 72-hour postoperative period (Fig 2); since PACU stay is only a small fraction of this 6-hour period, it seems unlikely to have influenced our findings, and d) the results of a sensitivity analysis, after excluding the first 6 postoperative hours, confirmed this statement.

Although the predominant perioperative analgesia regimen was opioid-based, the total amount of opioids (in intravenous morphine equivalents) consumed by our patients in the first 48 hours postoperatively, was similar to that observed in other bariatric surgery populations [37]. Dexamethasone and non-opioid analgesics that were given either intraoperatively or in the postoperative period, might have had the potential to affect postoperative pain and /or opioid consumption; however their effects cannot be considered as confounding the relationship between exposure (nocturnal recurrent hypoxemia in preoperative polysomnography) and outcomes (postoperative pain and opioid consumption) because a confounding variable, by definition, temporally occurs before the exposure [38]. Nevertheless, adjusting our analysis for the use of intraoperative analgesic medications, including dexamethasone, and for postoperative non-opioid analgesics (ketorolac, acetaminophen, and aspirin) did not change our findings. Therefore, we have not included those variables in our final regression model.

In conclusion, we found that recurrent nocturnal hypoxemia in bariatric surgery patients was significantly associated with decreased opioid consumption for postoperative pain management. To the extent that these findings are confirmed by prospective investigations in various surgical settings may indicate that in patients suffering from OSA, nocturnal intermittent hypoxia should be considered as a determinant of postoperative opioid pharmacology.

## Supporting Information

S1 Dataset (SAS7BDAT)

S2 Dataset (CSV)

S1 Fig Boxplots of the percentage of total sleep time spent at SaO_2_ < 90% and minimum nocturnal SaO_2_ for the 218 patients included in the analysis. The first quartile, median, and third quartile comprise the boxes; whiskers extend to the most extreme observations within 1.5 times the interquartile range of the first and third quartiles, respectively; points outsides these whiskers are displayed individually. SaO_2_ = arterial oxygen saturation by pulse.
(TIF)
