# Supplementary figures and images for: Chronic Intermittent Hypoxia Is Independently Associated with Reduced Postoperative Opioid Consumption in Bariatric Patients Suffering from Sleep-Disordered Breathing
Source: PLoS One. 2015 May 26;10(5):e0127809. doi: 10.1371/journal.pone.0127809 (PMC4444020; doi:10.1371/journal.pone.0127809)

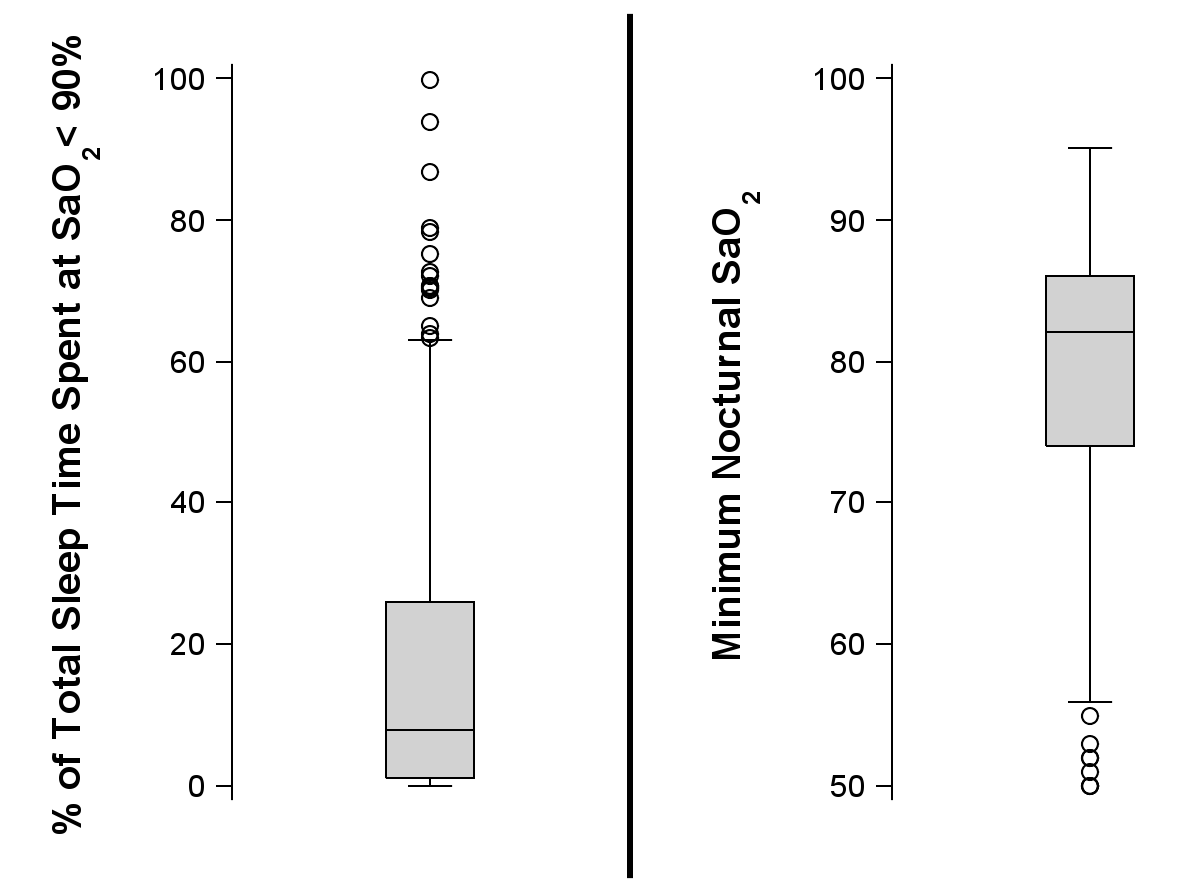

Supplement: S1 Fig — The first quartile, median, and third quartile comprise the boxes; whiskers extend to the most extreme observations within 1.5 times the interquartile range of the first and third quartiles, respectively; points outsides these whiskers are displayed individually. SaO2 = arterial oxygen saturation by pulse. (TIF) [file pone.0127809.s003.tif]
